# A Case of Hypertrophy of the Left Ventricle, Complicated with Pleurisy, &c.

**Published:** 1854-06

**Authors:** J. D. Mitchell

**Affiliations:** Darwin, Illinois


### ART. II.—A CASE OF HYPERTROPHY OF THE LEFT VENTRICLE, COMPLICATED WITH PLEURISY, &c.—PARACENTISIS THORACIS.

BY J. D. MITCHELL, M. D., OF DARWIN, ILLINOIS.

The patient, Henry Taylor, aged twenty-seven years, is a farmer by occupation, is said to have had bad health from his childhood. Had dropsy of the peritoneum at the age of eight or nine years, and has had a very large abdomen since that time. He has had several attacks of pneumonia and pleurisy. He first came under my care October 7th, 1853, for an attack of bilious or acute dysentery. This was somewhat obstinate, and did not yield to treatment till the third week. During this period, and even when he was not able to walk the floor from debility, he had a very full, hard and strong pulse, one hundred per minute. His spleen was much enlarged, and from accounts, has been in this condition for several years.

November 11, he again consulted me for anasarca of the inferior extremities. At this time he had the same full, hard, strong and frequent pulse as before, with strong pulsations of the heart over the precordial region. The anasarca disappeared in the course of three weeks, under the use of blood-letting, hydrogogue purgations, diuretics and an occasional mercurial; so that he was able to do considerable day labor through the month of December and a part of January; but frequently told me that if he exerted himself to any extent, he "was short of breath," and that it made his "heart beat." At the time he consulted me for anasarca, my attention was first directed to his heart as a seat of disease, and I had some fears that he was laboring under hypertrophy of the left ventricle; but as there were other derangements, such as enlargement of the spleen, scanty urine and torpid liver, sufficient to account for the anasarca, paid but little attention to the heart symptoms.

On the 20th of January, 1854, after being exposed to rain and cold the day before, he was taken with chilliness and pain beneath the left axilla and in the left shoulder, of a somewhat acute and lancinating character. This continued without any treatment, except of a simple nature used by the family, until the 26th when I was requested to visit him, a distance of three miles. As I could not see him on account of professional engagements, at my request W. J. CHENOWETH, M. D., who was in my office when I examined the patient last, visited him on the 26th and 28th. I suggested to the doctor the propriety of making a careful exploration of the chest, as the probability was that he was laboring under chronic pleurisy. He reported to me his conviction of the existence of fluid in the left pleura. The patient was freely cupped over the seat of pain, and was attended with decided relief. Dover's powder was given, to procure rest, at night, and small doses of tarter emetic through the day.

January 30th, I visited him, and found him laboring under the following symptoms and physical signs: Pulse full, hard and strong, one hundred and ten per minute; tongue covered with a thick yellowish fur, and quite dry; superficial veins distended; face somewhat of a turgid appearance, and a livid cast. He is hoarse and does not speak above a whisper; cough troublesome and hoarse; expectoration scanty and tough; breathing short and labored, and much increased by the slightest exertion. Abdomen large, spleen much enlarged, urine high colored and scanty, but little pain on the left side.

*Physical signs.*—He lay on his back, with an inclination to the left side, and his shoulders elevated. The right shoulder was a little depressed in the sitting position, intercostal spaces partly obliterated. There was dullness on percussion, amounting to flatness, over the whole of the left side, even to the top of the chest. The right side elicits the natural sound, if any deviation from health, a little dull. On applying the ear to the left side, there were no sounds heard, except the pulsation of the heart near the spine and under the sternum. The vesicular murmur was marked and exaggerated on the right side. The pulsations of the heart could be heard and felt over the whole of the right side. No breathing movements on the left side, remarkably contrasting in this respect with the right. The left side measured one-half inch more than the right.

*Treatment.*—From the fact that there is a decided inflammatory tendency, and derangement of the secretions generally, and more especially of the digestive organs, I ordered, calomel 10 gr., Dover's powder 10 gr., to be followed in eight hours with the infusion of senna and sulph. magnesia; and repeated next day in the same manner. With the view of acting on the kidneys. the respiratory organs, and the exhalants generally, I ordered tart. emetic iv. grs., nit. potass. ʒii., cr. tartar ℥i., in water vi.; a table-spoonful every three hours. Dovers powder at night.

February 1. Symptoms much as before, with the exception that the pulse is some reduced in volume and frequency, and expectoration more free. The purgatives acted freely, and brought away a large quantity of dark green and feculent matter. He has perspired freely. Physical signs unaltered. The treatment was continued, omitting the mercurial. A large blister was placed over the left side, and the region of the spleen and adjoining thorax freely cupped.

February 3d. Pulse one hundred and ten, full, hard and strong. Tongue somewhat dry and coated. Ordered blood-letting xv℥., calomel iiigrs., Dover's powder viiigrs., one every four hours till four are taken. A large blister over the spleen and thorax. Antimonial mixture continued. The infusion of digitalis three times per day.

February 5. Pulse somewhat reduced in frequency and force; kidneys acting more freely; has been purged freely, and ejections of a dark-green cast; apex of the heart is felt at the right of the sternum; the lividity of the face and surface of the body is evidently increasing. The dyspnœa is also increasing. He is not able to help himself, and much fatigued from the slightest exertion. He is inclined to sleep most of the time, with some tendency to coma. There is no evidence that the fluid is being absorbed, and if relief is not had very soon he must die from a deficiency of oxygen in the blood. I consulted the patient and friends in reference to the operation of paracentisis thoracis, and expressed my opinion that he could not live long without the operation; and that though the operation might not save him, his prospects would be more favorable for recovery.

February 6th. Visited him in company with Dr. Chenoweth.

Symptoms as before; much the same course of treatment continued. We are of opinion that there is hypertrophy of the left ventricle, a large quantity of fluid in the cavity of the left pleura, and great enlargement of the spleen. We concur in the opinion that he should have the benefit of an operation. We were satisfied that if the many derangements of the system were the results of the probable existing hypertrophy, the operation would afford only temporary relief; but we were convinced that the fluid in the pleura was the result of separate and local inflammatory action.

February 8th. Yesterday the patient and friends gave their consent to have the operation performed to-day. The symptoms are much as they have been for some days past. The lividity of the face is slowly increasing, an increased tendency to coma, sleeps most of the time if not disturbed by his attendants, and when asleep is constantly picking and working with the bed clothing, rational when aroused, does not speak above a whisper, coughs but little. The apex of the heart is felt under the right nipple; pulse one hundred, full and strong; left side flat on percussion, and measures one inch more than the right side; intercostal spaces of the left side entirely obliterated, and cellular tissue injected.

The operation was performed in the presence of Dr. CHENOWETH, who assisted, and of several friends of the patient. As we had no trocar and canula, the instruments used were a sharp pointed scalpel and a female catheter. A small delicate leather was placed over the external orifice of the catheter, to act as a valve, which proved a good expedient to prevent the entrance of air into the chest during the flow of fluid. The spot selected, was the space between the fifth and sixth ribs, half way between the spine and center of the sternum. The wound externally was half an inch, and just large enough through the pleura to admit the catheter. Nothing was discharged till the catheter was introduced, when in the course of twenty-five or thirty minutes one gallon of fluid, some colored with blood, escaped. During the escape of fluid, the patient complained of sharp pains beneath the sternum; owing, most probably, to the tearing up of adhesions between the pericardium and pleura costalis, and the compressed lung and pleura, whilst those organs were assuming their natural position. His breathing improved from the time the fluid began to escape; and after all had escaped, it was easy, and but little accelerated. The apex of the heart was felt beating faintly to the left of the sternum, but a little higher up than natural. He appeared much refreshed and relieved after the operation; but his pulse remained unaltered.

*Treatment:*—Morphine and ipecac, to procure rest, and pil. hydrarg. 10 grs. at night. A table spoonful of the following was given three times a day:

| | | |
|---|---|---|
| ℞. | Iodide Potassa, | ʒi. |
| | Tr. mur. ferri, | ʒii. |
| | Tr. Iodine, | ʒii. |
| | Water, | ℥vi. |

Bitart. potass was ordered freely, and purgatives when necessary. The infusion of digitalis was continued up to the 15th, with the view of exciting the kidneys and controlling the action of the heart and arteries.

15th. There is evidently an accumulation of fluid in the peritoneum, but not very large in quantity. Percussion over the left side elicited the natural sounds, except a preternatural dullness over the heart. Right side probably natural, though I have thought there was a little dullness throughout its whole extent. Pulse as before,—cough not troublesome, and expectoration slight, —tongue clean. He has walked across the house several times, and sits up by the fire occasionally and is evidently gaining strength slowly.

20th. Symptoms as before,—pulse full, hard, and strong,--abdomen more swollen,—tongue clean.

24th. Patient died last night between 9 and 11 o'clock. He had, to all appearance, improved up to the evening of his death; was able to sit up by the fire frequently through the day, and the same evening sat up and ate his supper at the table, heartily. At nine he was as well as usual, and requested his attendants to retire, which they did, leaving him resting on his right side. At eleven he was found dead in the same position.

*Autopsy*, Twenty-four hours after death, as reported by myself and Dr. Chenoweth, and afterward compared. External appearance:—countenance natural, no discoloration of the body, abdomen much distended and fluctuating. On opening the thorax, both lungs were found adherent to the costal pleura almost their entire extent, only a small space under the seat of the operation being full. The pericardium was pushed towards the upper end of the sternum, and attached to it by firm adhesions. The right pleura contained about a pint of fluid blood, probably the result of position at death. The left pleura contained about half a pint of clear fluid. The surface of the lungs presented a reddened and highly congested appearance, and their substance throughout was of a dark red appearance, with here and there a drop of blood exuding from the cut surface. They were somewhat solid and of a hepatized appearance, but would yield and crepitate slightly on pressure. The pericardium contained between a pint and a quart of transparent fluid. The right auricle was much distended with fluid blood, which would not escape by changing the position of the heart. The left ventricle and auricle were entirely empty. The heart was removed and placed in water till the next day. The walls of the left ventricle measured from eight lines to one inch in thickness, and the columna carnea were much enlarged. On opening the right ventricle two fibrinous concretions, of a whitish color, firm and tough, three or four inches in length, and a line and a half in thickness, together weighing one drachm, were removed. They were taken either from beneath the tricuspid valves, or the auriculo-ventricular opening. As our attention was not directed to their vermicular appearance, we did not carefully observe their location ; but they were loose, affording no resistance on lifting them out; and as a small piece was afterwards removed from beneath the valves, the probability is the first two came from the same place. The valves were roughened and congested in appearance.

The peritoneum contained about one and a half gallons of transparent fluid. This membrane, with the exception of the portion covering the intestines, was covered with innumerable lumps or granules, from the smallest size to that of a pea. They were most numerous in the lower portion of the abdomen. The mesentery contained a few of these granules, with here and there a livid, elevated and roughened patch, from one to four lines in diameter. The spleen was very large, probably weighing eight pounds. The liver was small in size, and easily broken down. The great omentum was pushed up over the stomach into a roll. It was of a hard, rough, granular feel, and much thickened, measuring an inch in the thickest parts. It was perforated in two or three places.

*Remarks.*—I have reported the above case as it was recorded from day to day, so as to give as correct a view as possible of its complicated nature, omitting many things of less importance. The points of interest in this case are its remarkably complicated nature, and the relation the complications sustain to the hypertrophy of the heart. Through the whole course of the disease there was evidently inflammatory symptoms greater than would naturally proceed from the hypertrophy; so that it is reasonable to conclude that some of the morbid conditions were not the result of the heart disease. The fluid in the left pleural sack was evidently the result of inflamation of that membrane, and that, too, of an acute character, caused by exposure. It was from this fact that I thought best to evacuate the fluid, and as it did not re-accumulate, it is evident that this view was correct; though, if the other morbid conditions had been fully known at the time, it would have been deemed useless to operate. On account of the quantity of fluid in the pleura, it is not known at what time the pericardial effusion first existed. The congested and solidified appearance of the lungs resulted, most probably, from the force of the heart's action immediately before death. When did these fibrinous concretions accumulate? before or after death? They were of too firm a structure to have been formed after death. May they not in some way or other (probably mechanically) have been the immediate cause of death? It is probable that the disease of the peritoneum, with its granular deposits, was of long standing.

